# Recurrent ventricular arrhythmias and heart failure induced by osimertinib- a case report

**DOI:** 10.3389/fcvm.2024.1423647

**Published:** 2024-08-29

**Authors:** Jiangying Luo, Boda Zhou, Jing Yang, Hao Qian, Yutong Zhao, Fei She, Fang Liu, Ping Zhang

**Affiliations:** Department of Cardiology, Beijing Tsinghua Changgung Hospital, School of Clinical Medicine, Tsinghua University, Beijing, China

**Keywords:** osimertinib, QT interval, Torsade de Pointes, T wave alternans, heart failure, isoproterenol, case report

## Abstract

**Background:**

Osimertinib is a third-generation epidermal growth factor receptor (EGFR)-tyrosine kinase inhibitor that has become the first-line treatment for non-small cell lung cancer harboring EGFR mutations, with the potential risk of QT prolongation and heart failure. However, few cases have reported malignant ventricular arrhythmias. Here, we report a case of recurrent ventricular fibrillation (VF) and Torsade de Pointes (TdP) secondary to QT prolongation and heart failure induced by osimertinib.

**Case summary:**

A 70-year-old woman presented with chest tightness and dyspnea for 1 week and ventricular fibrillation upon admission, with a medical history of lung adenocarcinoma harboring an EGFR exon 21 p.L858R mutation. She was under osimertinib for 3 months. Electrocardiography after defibrillation suggested QTc prolongation (655 ms) and T wave alternans. Ultrasound cardiography displayed left ventricular ejection fraction (LVEF) of 29% and severe mitral regurgitation. Laboratory tests indicated elevated N-terminal pro-B-type natriuretic peptide and hypokalemia. Genetic testing suggested no pathogenic mutations. We considered acquired long QT syndrome and heart failure with reduced ejection fraction induced by osimertinib as the chief causes of ventricular arrhythmia and hypokalemia as an important trigger. Despite intubation, sedation, and the administration intravenous magnesium and potassium and lidocaine, the patient presented with recurrent TdP, which was managed by a low dose of isoproterenol (ISO, 0.17 ug/min). An implantable cardioverter defibrillator was declined. The patient is surviving without any relapse, with QTc of 490 ms and LVEF of 42% after a 6-month follow up.

**Conclusion:**

Regular monitoring is required during osimertinib administration, considering the risk of life-threatening cardiac events, such as malignant arrhythmias and heart failure. ISO, with an individual dose and target heart rate, may be beneficial for terminating TdP during poor response to other therapies.

## 1 Introduction

Osimertinib is a third-generation epidermal growth factor receptor-tyrosine kinase inhibitor (EGFR-TKI) that has become the first-line treatment of non-small cell lung cancer (NSCLC) harboring EGFR mutations (exon 19 deletion and exon 21 L858R). Osimertinib has been strongly associated with QT prolongation (2.7%) and heart failure (∼1.4%–2.4%) (1). The most severe outcome of QT prolongation is Torsade de Pointes (TdP), which is often self-terminated, but may degenerate into ventricular fibrillation (VF) and cause sudden cardiac death (SCD). No fatal cardiac adverse events (AEs) related to osimertinib have been reported in clinical trials. The acute management of drug-induced QT prolongation and TdP includes the discontinuation of offending drugs, defibrillation if necessary, administration of intravenous magnesium, correction of electrolyte abnormalities, and overdrive pacing or isoproterenol (ISO) to increase the heart rate (HR) and shorten the QTc interval (2). Here, we report a case of osimertinib-induced QT prolongation and heart failure, accompanied by recurrent VF and TdP in a patient being treated for NSCLC.

## 2 Case report

### 2.1 Case presentation

**Figure 1:**
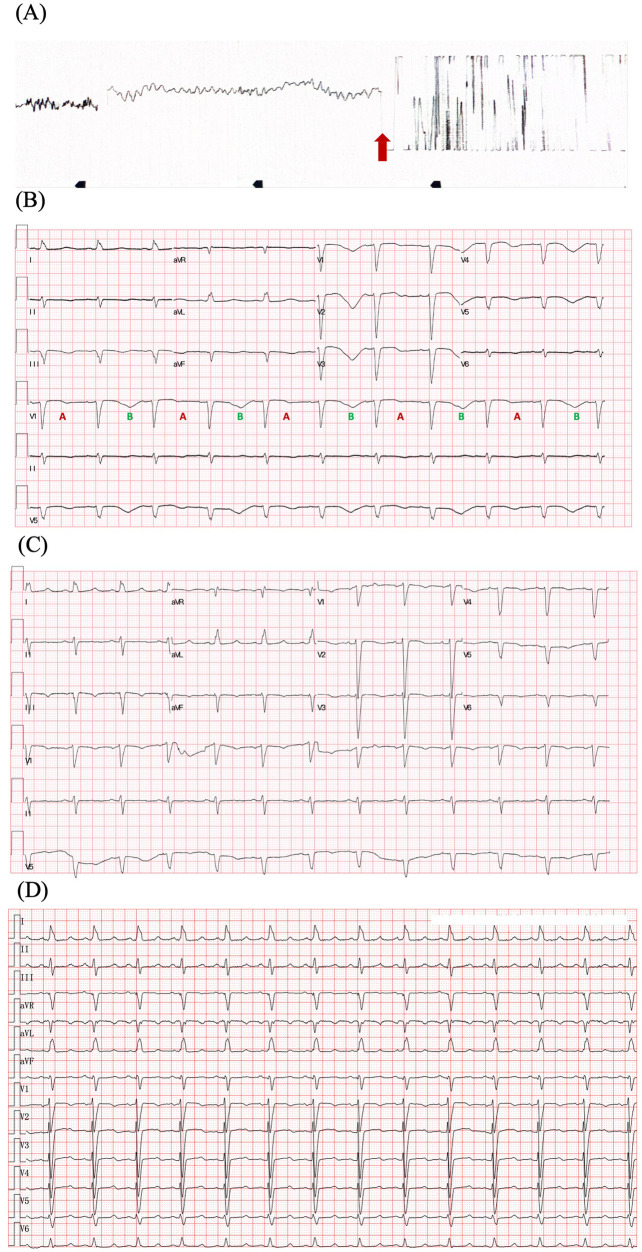
ECGs of the patient. **(A)** ECG illustrating ventricular fibrillation and defibrillation (red arrow). **(B)** ECG after defibrillation displays sinus rhythm, QTc prolongation (655 ms), and T wave alternans. The distinct beat-to-beat alternation of the T-wave structure with a repeating ABABAB pattern is observed in lead V1. **(C)** ECG before discharge displays QTc 509 ms and improved T-wave morphology. **(D)** ECG after 6 months follow-up displays QTc 490 ms. Heart rate correction of QT interval was performed by Bazett's formula. ECG, electrocardiography.

**Figure 2:**
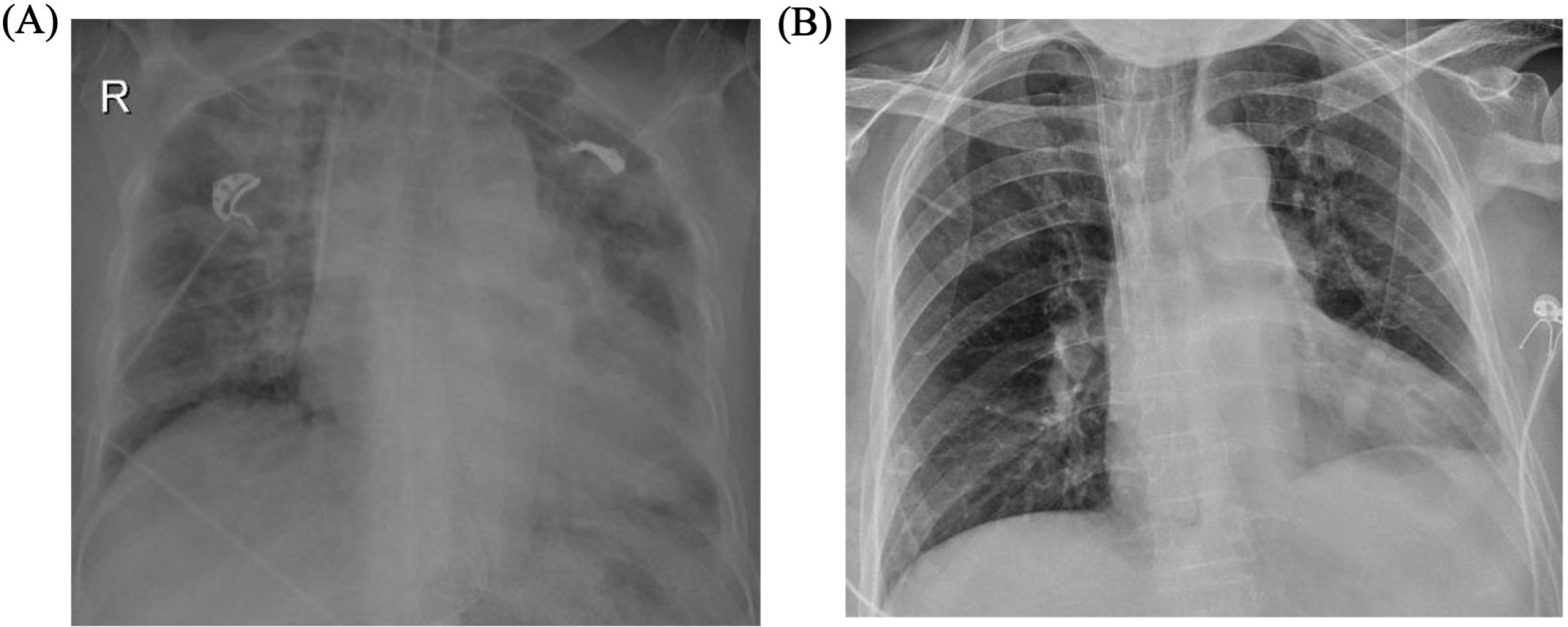
CXR on admission **(A)** and before discharge **(B)** CXR, chest x-ray.

A 70-year-old Chinese woman was admitted to our hospital with a chief complaint of chest tightness and dyspnea for 1 week. Three months ago, she was diagnosed with lung adenocarcinoma harboring an EGFR exon 21 p.L858R mutation. She underwent left upper lobectomy and mediastinal lymphadenectomy with video-assisted thoracoscope and started osimertinib 80 mg once daily at another hospital. The patient was a nonsmoker and had no medical history of cardiovascular diseases. Physical examination suggested a blood pressure (BP) of 125/80 mmHg and HR of 85 bpm, moist crackles in both lungs, and bilateral lower limb edema. She suddenly developed VF during the visit, which was treated with 200-J shock twice (Figure 1A). Electrocardiography (ECG) after defibrillation demonstrated QTc prolongation (655 ms, QTc was performed using the Bazett's formula) and T wave alternans (TWA), which manifested as a beat-to-beat alternation of the T wave structure with a repeating AB pattern annotated in lead V1 (Figure 1B). Laboratory tests indicated high levels of the N-terminal pro-B-type natriuretic peptide (NT-proBNP) (16,674 pg/ml, cut-off value of 125 pg/ml), high-sensitivity cardiac troponin (0.275 ng/ml, cut-off value of 0.014 ng/ml), and hypokalemia (2.98 mmol/L, cut-off value of 3.55 mmol/L). The remaining blood test results were normal. Ultrasound cardiography (UCG) suggested left ventricular (LV) dilatation [left ventricular end-diastolic diameter (LVEDD) of 54 mm], LV systolic dysfunction (LVEF of 29%), and severe mitral regurgitation (MR). Chest x-ray (CXR) displayed bilateral diffuse infiltration, mild left hydrothorax, and enlarged heart shadow (Figure 2A).

**Figure 3:**
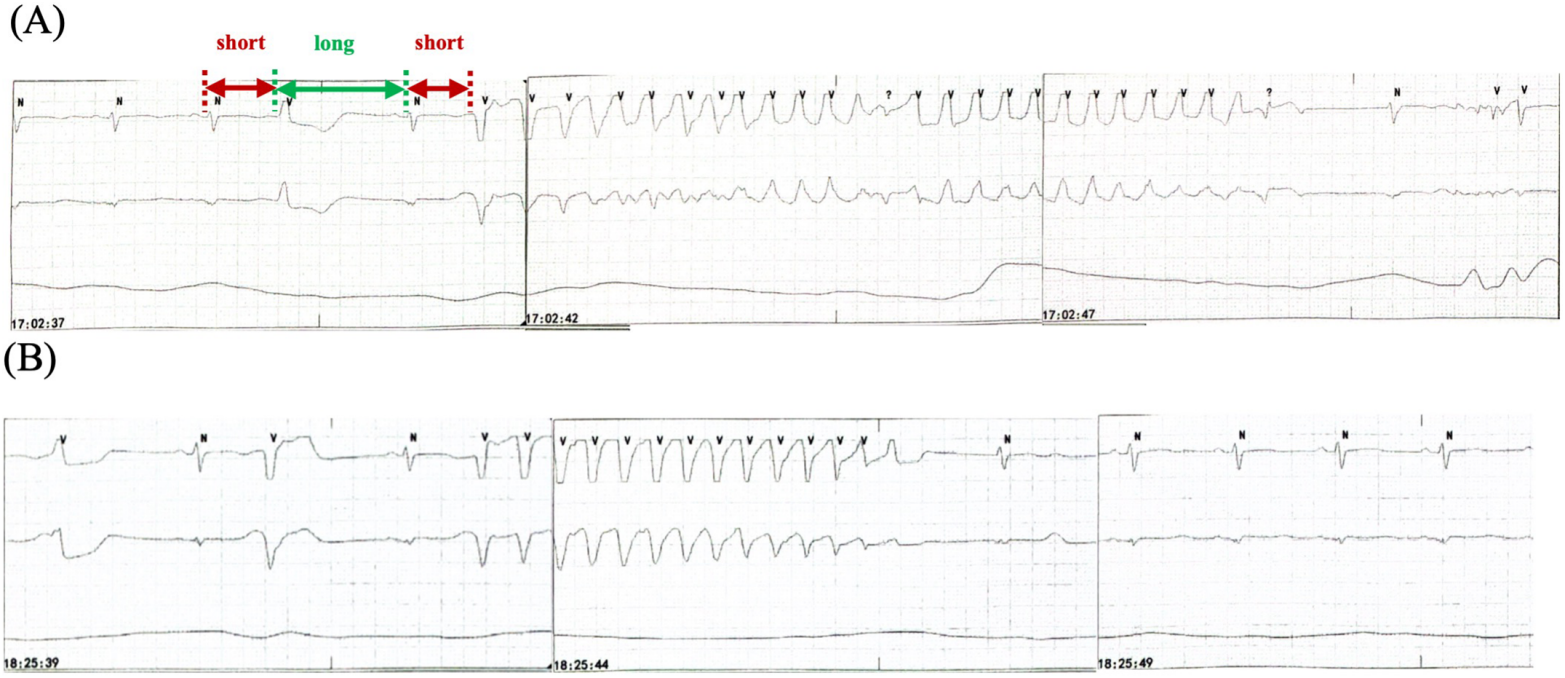
Recurrent TdP. **(A)** TdP with a “short-long-short” pattern of R-R cycles (red and green arrows) before intravenous ISO administration. **(B)** TdP recurrence when the HR decreases to the baseline level after terminating ISO. TdP disappears for 90 min with HR >60 bpm because of the use of ISO. HR, heart rate; ISO, isoproterenol; TdP, Torsade de Pointes.

The patient underwent intubation and sedation because of hemodynamic instability caused by recurrent ventricular arrhythmias (VAs), and received intravenous magnesium and potassium supplement, which maintained the electrolyte balance rapidly (K^+^ 4–4.5 mmol/L, Mg^2+^ 1.0 mmol/L). Diuretic was administered to reduce the capacity load. QTc shortened to 558 ms and TWA disappeared after correcting electrolyte disturbance (Supplementary Figure S1). However, the patient presented with recurrent TdP (Figure 3A). The patient had no family history of dilated cardiomyopathy, ventricular arrhythmias (VAs), syncope, and sudden cardiac death. Coronary angiography ruled out obstructive artery disease 1 year ago. Her baseline ECG and UCG were normal before initiating osimertinib, with QTc of 451 ms, LVEDD of 49 mm, and LVEF of 60% (Supplementary Figure S2). There were no records of cardiac follow-up during the treatment of osimertinib. The genetic testing did not indicate pathogenic mutations. Thus, we considered acquired long QT syndrome (aLQTS) and HFrEF induced by osimertinib as the chief causes of VAs and hypokalemia as an important trigger.

**Figure 4:**
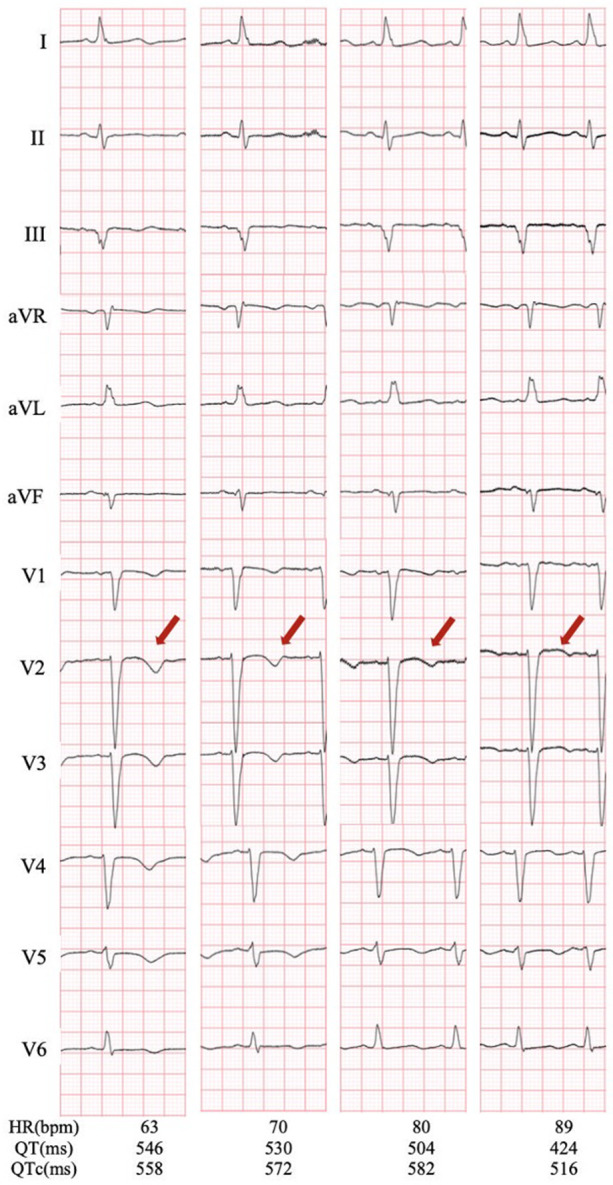
ECG during intravenous ISO administration. Shortness of QTc from 558 ms to 519 ms with HR from 63 bpm to 89 bpm is observed, along with improved T wave morphology in the precordial leads from inversion to upright (red arrow). ISO, isoproterenol; ECG, electrocardiography.

Osimertinib was discontinued. Lidocaine, which may shorten QTc interval by blocking late-sodium current, was administered but was proven ineffective. The family refused temporary transvenous pacing, and we finally agreed on intravenous ISO. Upon administering ISO at 0.5 ug/min [recommended ISO dose is 0.5–5 ug/min and target HR is 90–110 bpm per the American Heart Association/American College of Cardiology guideline (2)], the HR increased from 60 bpm to 90 bpm. Continuous ECG tests suggested shortness of QTc from 558 ms to 519 ms and improved T wave morphology (Figure 4). The recurrent TdP was terminated. However, we observed a gradual decline in systolic BP (SBP) from 120 mmHg to 85 mmHg, which was attributed to the worsened MR and decreased cardiac output index with faster HR. Thus, we terminated ISO immediately. The SBP recovered gradually, but TdP recurred when the HR decreased to 60 bpm, thus confirming the efficacy of ISO (Figure 3B). To prevent VAs and ensure hemodynamic stabilization simultaneously, we re-titrated to a low dose (0.17 ug/min) of ISO and attained stable SBP and quiet ECG monitoring with an HR of approximately 80 bpm. The TdP did not relapse. NT-proBNP gradually declined to 491 pg/ml, and repeated CXR suggested improved effusion and smaller heart shadow (Figure 2B). The patient was extubated on day 7, and ISO was terminated on day 9. Before discharge on day 21, repeated ECG suggested QTc of 509 ms and improved T wave morphology (Figure 1C). Repeated UCG indicated normal LVEDD, no MR, and LVEF of 39%.

The QTc did not recovered to normal level. Considering the history of recurrent VAs, we recommended implantable cardioverter defibrillator (ICD) for the secondary prevention of SCD. However, the patient and family refused. We recommended purchasing automated external defibrillator (AED) if needed. The patient was discharged on medical therapy consisting of potassium magnesium aspartate tablets, perindopril, spironolactone, dapagliflozin. Osimertinib was replaced by chimeric antigen receptor immunotherapy. After a 6-month follow-up, the patient did not present with any symptoms. QTc interval shortened to 490 ms (Figure 1D) and LVEF increased to 42%.

## 3 Discussion

Osimertinib is an oral, third-generation, irreversible EGFR-TKI that has become the standard of care for the first-line treatment of NSCLC harboring EGFR mutations. Common side effects of Osimertinib include diarrhea, rash, nausea, decreased appetite and paronychia. Osimertinib has also been strong associated with QTc prolongation and cardiac failure relative to other EGFR-TKIs (1). A research based on data from the U.S. Food and Drug Administration Adverse Event Reporting System (FAERS) demonstrated that the reporting OR (ROR) (95% CI) for heart failure and QTc prolongation in comparing osimertinib vs. other EGFR-TKIs was 2.2 (1.5–3.2) and 6.6 (3.4–12.8) (3). Cardiac failure was the most common cardiac AE caused by Osimertinib, followed by QTc prolongation. In this study, 1.3% patients developed QTc prolongation, with median time to onset of 23 days (IQR: 14–55), 2.3% patients developed cardiac failure, with median time to onset of 29 days (IQR: 16.5–95.5). A post-marketing surveillance study of Osimertinib in Japan suggested that the incidence of Grade 3 or higher QTc prolongation (QTc >501 ms or an increase from baseline >60 ms) occurred in only 0.1% and other Grade 3 or higher cardiac AEs in 0.8% (4).

The mechanisms of osimertinib-induced QTc prolongation may associate with potassium channels, i.e., Kv1.3 (5), Kv10.1 (6), Kv4.3 (7), Kir2.3 (8) and Kv11.1 (9), which plays an important role in repolarization. Osimertinib has been proved as an inhibitor of Nav1.5 channel in a concentration-dependent manner, and slightly inhibited the current of L-type Ca^2+^ channels in a concentration-dependent manner in acutely isolated rat ventricular myocytes (9). Besides, animal models suggested that inhibition of the PI3K pathway may be related to QT prolongation (10). The etiology of osimertinib-induced cardiac failure is unclear. It may be similar to trastuzumab, a monoclonal antibody against human EGFR (HER2) (11). Besides, osimertinib has been reported to reduce cardiomyocytes viability, increase cardiac BNP expression, and induce apoptosis directly (12).

No fatal cardiac AEs were reported in clinical trials. However, in the real-world setting, TdP due to QTc prolongation were reported in a limited number of cases (Supplementary Table S1), among which 2 cases were accompanied by cardiac failure. Bian et al. (13) reported the first case of an 85-year-old man with advanced NSCLC had TdP induced by osimertinib (80 mg once daily), moxifloxacin and hypokalemia (2.94 mmol/L), accompanied by impaired LVEF (41%). TdP was controlled and QTc shortened to 496 ms after discontinuation of offending agent, magnesium and potassium supplementation and intravenous lidocaine. But the patient experienced decreased blood pressure and pulse oxygen and was discharged without invasive rescue measures. Ikebe et al. (14) reported an 80-year-old Japanese woman developed TdP and cardiac failure (LVEF 35%) induced by osimertinib (80 mg once daily). TdP was controlled by electrocardioversion. QTc returned to 464 ms at discharge and LVEF improved to 62% at 4 months after osimertinib discontinuation. However, the patient did not receive any other chemotherapy and died of cancer progression after 15 months.

In this case, the patient demonstrated QTc prolongation from 451 ms to 655 ms and decline of LVEF after using osimertinib 3 months. Furthermore, ECG after defibrillation demonstrated a repetitive ABABAB pattern in the morphology and amplitude of the T wave, termed TWA. It was associated with fatal arrhythmia caused by spatiotemporal heterogeneity of repolarization. Ischemia and genetic mutations did not occur. She developed recurrent TdP despite osimertinib discontinuation, correcting electrolyte disturbance and intravenous lidocaine. Overdrive pacing by a pacemaker were reported to control TdP successfully in two cases (15, 16), which was refused by the family. Similar to overdrive pacing, ISO infusion can speed up the heart, shorten the QTc interval and may terminate TdP effectively (2). To our knowledge, this is the first case reporting ISO in the acute management of osimertinib-induced VAs. An ISO dose of 0.5 ug/min–5 ug/min is recommended; the target HR of 90 bpm–110 bpm or higher may be used if TdP recurs per the guideline (2, 17). In this case, we used an ISO dose of 0.5 ug/min, which is the lowest recommended level. TdP disappeared; however, SBP declined. We titrated the infusion rate to 0.17 ug/min with ideal HR of approximately 80 bpm, which was lower than the target HR. The SBP recovered, and TdP did not recur. Higher HR caused the deterioration of hemodynamics because of severe MR. Furthermore, the baseline HR before ISO, approximately 60 bpm, was not defined as marked bradycardia in clinical practice, which resulted in QT prolongation and TdP. Nonetheless, ISO was beneficial. Thus, the baseline HR before initiating ISO and the ideal target HR had differences, thereby warranting careful observation and treatment.

Patients with heart failure are often managed by discontinuing osimertinib and with guideline-directed medical therapy, including renin-angiotensin system inhibition, beta-blockers, mineralocorticoid reception antagonists, and sodium-glucose cotransporter 2 inhibitors, as well as adjunctive therapies, such as loop diuretics (1). It is unclear whether patients who develop cardiomyopathy while on osimertinib can be rechallenged after the recovery of ejection fraction. In this case, the patient received diuretics during the hospitalization to reduce the capacity load. The repeated CXR suggested improved effusion; the LVEF recovered from 29% to 39% at discharge. The discharge medicines included perindopril, spironolactone, and dapagliflozin. We did not prescribe beta-blocker because of VT/VF recurrence during decreased HR. The LVEF increased to 42% after 6 months follow-up.

ICD is the only useful method to prevent SCD. However, its role in aLQTS after cardiac arrest remains controversial. Mönnig et al. (18) followed up patients with high-risk aLQTS and ICD; ICD shocks were common (44%) regardless of the underlying cause or structure of heart disease. Moreover, most shocks occurred during normal daily conditions (58%) or at rest (26%) at a mean follow-up of 84 months. ICD may be beneficial even in acquired proarrhythmia; however, it was refused by the patients. AED may be an alternative.

## 4 Conclusion

Regular monitoring is required during osimertinib treatment, considering the risk of life-threatening cardiac events, such as malignant arrhythmias and heart failure. ISO, with an individual dose and target HR, may be beneficial for terminating TdP for no response to other therapies.

## Data Availability

The original contributions presented in the study are included in the article/Supplementary Material, further inquiries can be directed to the corresponding author.
