# Supplementary material for: Recurrent ventricular arrhythmias and heart failure induced by osimertinib- a case report
Source: Front Cardiovasc Med. 2024 Aug 29;11:1423647. doi: 10.3389/fcvm.2024.1423647 (PMC11390421; doi:10.3389/fcvm.2024.1423647)
Supplement: Supplementary file 1 [file Datasheet1.docx]

Supplementary Material


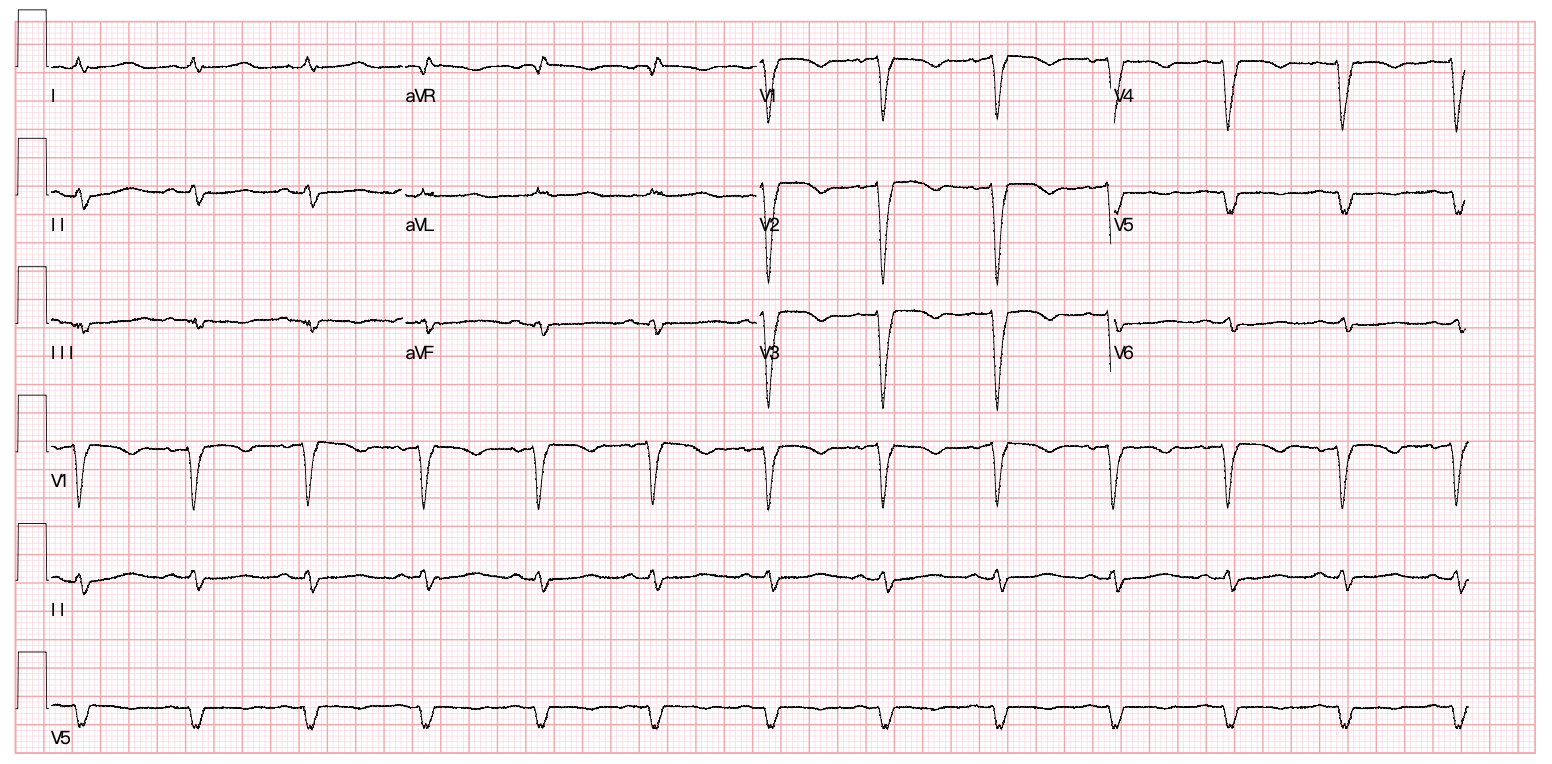


**Supplementary Figure 1.** ECG after correcting electrolyte disturbance (maintaining K^+^4-4.5 mmol/L, Mg^2+^ 1.0 mmol/L) displays QTc 558ms and no T-wave alternans

Abbreviation: ECG, electrocardiography

**
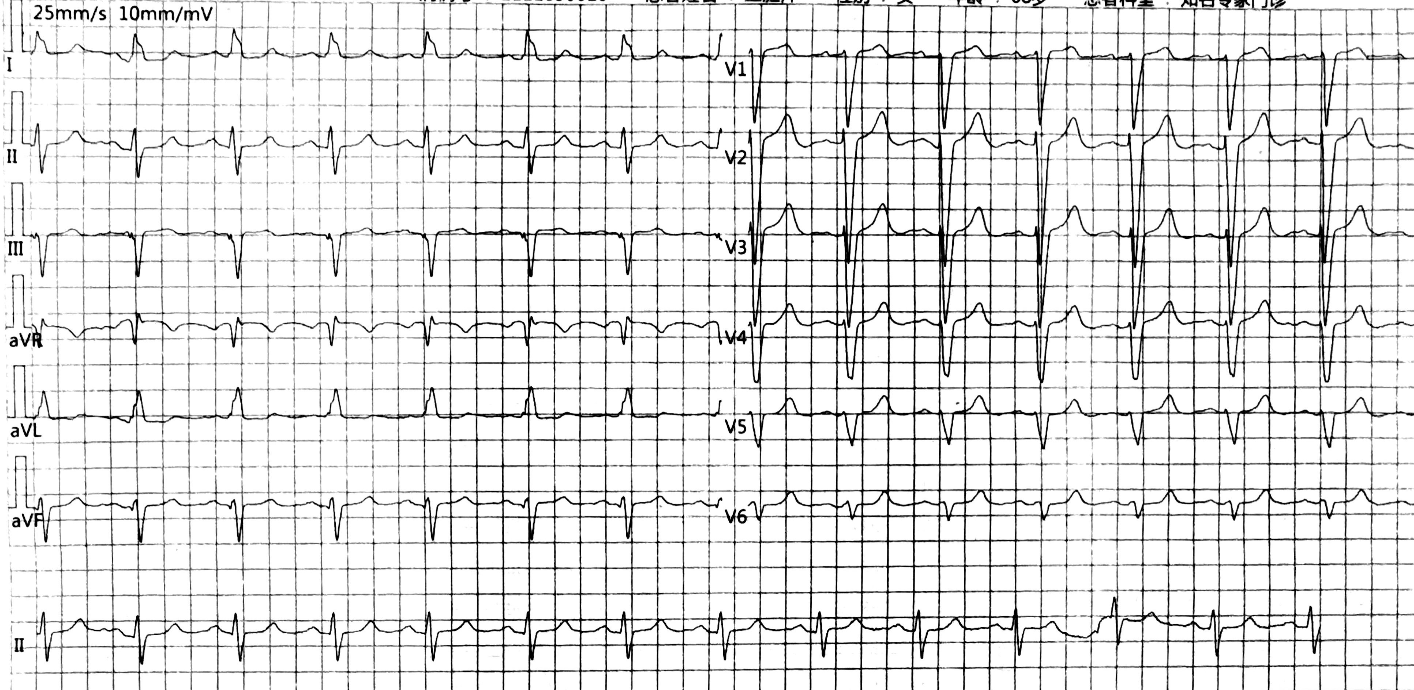
Supplementary Figure 2.** Baseline ECG before initiating Osimertinib displays left anterior fascicular block, with QTc of 451ms and QRS of 119ms.

Abbreviation: ECG, electrocardiography

**Supplementary Table 1. Summary of Osimertinib-induced VAs cases reported.**

| Author [Ref] | Age, sex | Osimertinib treatment time | QTc interval | VAs | LVEF | Suspicious concomitant risk factors | treatment | Follow-up |
| --- | --- | --- | --- | --- | --- | --- | --- | --- |
| Matsuura et al. (18) | 60, F | 2 months | 532ms | TdP | ND | General anesthesia; perioperative stress. | Osimertinib discontinued, magnesium supplementation. | QTc shortened to 475ms at discharge. |
| Ikebe et al. (13) | 84, F | 2 months | 524ms | TdP | 35% | No | Osimertinib discontinued, cardioversion, enalapril maleate, bisoprolol fumarate. | QTc shortened to 464ms at discharge. LVEF increased to 51% after 8 months follow-up.  She died of cancer progression after 15 months. |
| Bian et al. (12) | 85, M | 6 months | 647ms | TdP | 41% | Hypokalemia; Moxifloxacin | Osimertinib and moxifloxacin discontinued, magnesium and potassium supplementation, intravenous lidocaine. | QTc shortened to 496ms after 91 hours. He experienced decreased blood pressure, pulse oxygen saturation, and was unconscious, and was discharged without invasive salvage measures. |
| Kaira et al. (19) | 72, F | 3 months | >600ms | VF | ND | ND | Osimertinib discontinued; Cardiopulmonary resuscitation. | ND |
| Zhang et al. (14) | 60, F | 17 months | 640ms | VT, TdP | Normal | No | Osimertinib discontinued, magnesium and potassium supplementation, oral propranolol, Overdrive pacing by temporary pacemaker (110 bpm) | Osimertinib was replaced by gefitinib (250 mg QD). QTc decreased to 477ms. The 3- and 6-month follow-ups showed good recovery and normal ECG results. |
| Zhang et al. (15) | 68, F | 2 months | 532ms | TdP | Normal | Litsea Cubeba | Cardiopulmonary resuscitation; magnesium and potassium supplementation, intravenous lidocaine, overdrive pacing by a pacemaker (100 bpm) | QTc did not return to normal (QTc 528ms). |
| Our case | 70, F | 3 months | 655ms | VF, TdP | 29% | Hypokalemia | Defibrillation, sedation, intubation, supplementation of potassium and magnesium, intravenous lidocaine intravenous isoproterenol (80bpm); spironolactone; dapagliflozin. | QTc shortened to 490ms and LVEF increased to 42% after 6 months follow-up |

Abbreviation: VAs, ventricular arrhythmias; LVEF, left ventricular ejection fraction; TdP, torsade de pointes; ND, not documented; VF, ventricular fibrillation; VT, ventricular tachycardia; bpm, beats per minute; ECG, electrocardiography.
